# Human Keratin Matrices Suppress Matrix Metalloproteinase Activity to Support Wound Healing

**DOI:** 10.3390/ijms252312898

**Published:** 2024-11-30

**Authors:** Allison N. Ramey-Ward, Shakesia Smith, Howard Walthall, Thomas H. Barrows

**Affiliations:** ProgenaCare Global, LLC, Marietta, GA 30067, USA

**Keywords:** keratin, biomaterial, wound healing, MMP, enzyme activity

## Abstract

Elevated protease activity is a hallmark of non-healing chronic wounds. Though multiple biomaterials exist that are successful in treating wounds, their roles in modulating the enzymatic environment of the wound are only beginning to be elucidated. Because keratin has long been known to be resistant to degradation by most enzymes, we studied a keratin biomaterial, the human keratin matrix (HKM), in the presence of enzymes identified to contribute to wound chronicity: neutrophil-derived elastase (NE), matrix metalloproteinase 1 (MMP-1), and MMP-9. Upon finding the suppression of MMP-9 activity in the presence of HKM without reducing enzyme protein levels, we further studied the ability of HKM to bind metal ions in the wound and showed the reduction of Zn^2+^ ion concentration in the presence of HKM. Finally, because of the enzyme resistance of keratin and the suppression of wound enzymes, we demonstrated that HKM was durable in the wound environment, and did not degrade in wound healing efficacy when left in place for two weeks compared to one week in a diabetic mouse model of wound non-healing. In this way, we show HKM is a unique and effective biomaterial for the treatment of chronic wounds through the modulation of wound MMP activity.

## 1. Introduction

Chronic wounds are a growing concern in global healthcare, estimated to afflict millions of patients worldwide [1]. These ulcers, which fail to close normally over an extended period of time, typically three months or more [2], often occur in correlation with other growing public health concerns, such as diabetes, obesity, and aging [3]. Because of this, increasing research efforts have been dedicated both to understanding the causes of wound chronicity and developing new technologies to promote healing.

Recently, elevated protease activity has emerged as a contributing factor to wound chronicity. In normal wound healing, enzymes, such as matrix metalloproteinases (MMPs) and serine proteases, play an important role in tissue repair and remodeling [4,5]. However, the activity level of these enzymes can become dysregulated, leading to wound nonhealing by breaking down newly formed tissue matrix [6,7]. MMP-1, MMP-9, and neutrophil-derived elastase (NE) have all been shown to be elevated in chronic wounds, further supporting that the elevated protease activity of these enzymes inhibits healing [8,9]. Collagenases and gelatinases, such as MMP-1 and MMP-9, respectively, can break down newly formed extracellular matrix proteins, like collagen and laminin [10,11], and NE, as the name suggests, targets elastin.

Another abundant protein in the skin that is important in wound healing is keratin. The structure of keratin contains multiple covalent crosslinks in the form of disulfide bonds that make it highly resistant to enzymatic degradation. Indeed, collagenase, elastase, trypsin, and other common proteases in the body cannot easily break down keratin [12]. Further work has demonstrated that keratin is not only enzyme-resistant but can also reduce the activity of MMPs [13].

As such, keratin has recently been investigated as a biomaterial to support wound healing. Products derived from sheep’s wool keratins or human hair keratins are of particular interest due to the abundance of these protein sources. These keratins share some structural homology with those found in the skin, and can be easily formed into biomaterials with mechanical durability, biocompatibility, and demonstrated healing efficacy [14]. A human keratin matrix (HKM) was recently shown to improve wound healing in vivo in a delayed wound healing model compared to other biomaterials and may modulate the inflammatory environment produced by cells in the wound bed [15]. Recent, expanding clinical data also show that HKM improves healing in a variety of chronic wound types, such as diabetic lower extremity wounds and venous leg ulcers [16,17,18]. These successes in chronic wound healing raise the question of the role of HKM in managing elevated protease activity in chronic wounds. However, because of the crosslinked nature of keratin in HKM, additional study is needed to determine if keratin in this form is similarly functional to other keratins to inhibit protease activity.

In this work, the interaction of HKM with proteases involved in chronic wound elevated protease activity was investigated in vitro. Further, the mechanism of keratin inhibition of MMPs was studied in HKM. Finally, the ability of HKM to maintain its effect over time in a wound environment was evaluated in vivo in a diabetic mouse chronic wound model.

## 2. Results

### 2.1. HKM Suppresses MMP Activity but Not Elastase Activity

Because keratin is resistant to degradation by a broad variety of proteases, including MMPs and serine proteases, we first evaluated the ability of HKM to suppress the activity of several such enzymes known to be elevated in chronic wounds: NE, MMP-1, and MMP-9. Pathologically relevant levels of these enzymes were each incubated with HKM or an appropriate enzyme inhibitor (elastatinal for NE, or N-Isobutyl-N-(4-methoxyphenylsulfonyl)glycyl hydroxamic acid (NNGH) for MMPs). Proteolytic activity was then measured for each enzyme over time and quantified as the reaction rate during the linear phase of the reaction.

**Figure 1:**
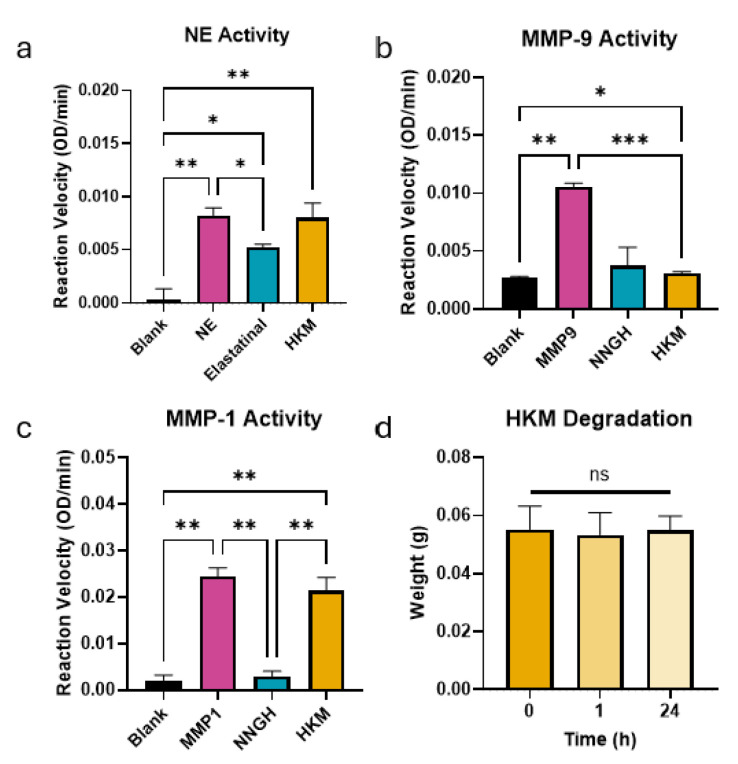
Human keratin matrix (HKM) reduces the activity of matrix metalloproteinase 9 (MMP-9) and resists degradation. (**a**) Bar graph showing the effect of HKM on neutrophil-derived elastase (NE) activity compared to the NE inhibitor elastatinal. (**b**) Bar graph showing the effect of HKM on MMP-9 activity compared to the MMP inhibitor N-Isobutyl-N-(4-methoxyphenylsulfonyl)glycyl hydroxamic acid (NNGH). (**c**) Bar graph showing the effect of HKM on MMP-1 activity compared to the MMP inhibitor NNGH. (**d**) Bar graph showing the weight of bulk HKM samples incubated with MMP-9 was not changed during the duration of the enzymatic activity assays (1 h) or up to 1 day after. Bars represent mean ± standard deviation of n = 3 independent replicates. * *p* < 0.05, ** *p* < 0.01, *** *p* < 0.001; ns = not significant by Brown–Forsythe and Welch analysis of variance (ANOVA) with Dunnett’s multiple comparisons.

HKM did not reduce the activity of NE compared to the enzyme alone but was also not significantly different than the reaction rate of NE incubated with elastatinal (Figure 1a). MMP-9 activity, however, was reduced by 70% upon incubation with HKM. This reduction was not different from that observed from incubation with NNGH, showing a significant effect of HKM in reducing MMP-9 activity (Figure 1b). Interestingly, MMP-1 activity only showed a slight but non-significant reduction in activity of 13% (Figure 1c). These data further highlight the effects of HKM on enzymatic activity, suggesting it is effective in MMPs but not serine proteases.

Samples of HKM were then incubated for longer to see if the reduced activity of MMP-9 in solution was related to the degradation of the keratin material. However, HKM weight was not changed over 24 h (Figure 1d). This demonstrates that HKM is resistant to degradation by MMP-9, and does not reduce MMP-9 activity in solution by acting as a preferred substrate for the enzyme.

### 2.2. HKM Does Not Reduce MMP Enzyme Levels in Solution

**Figure 2:**
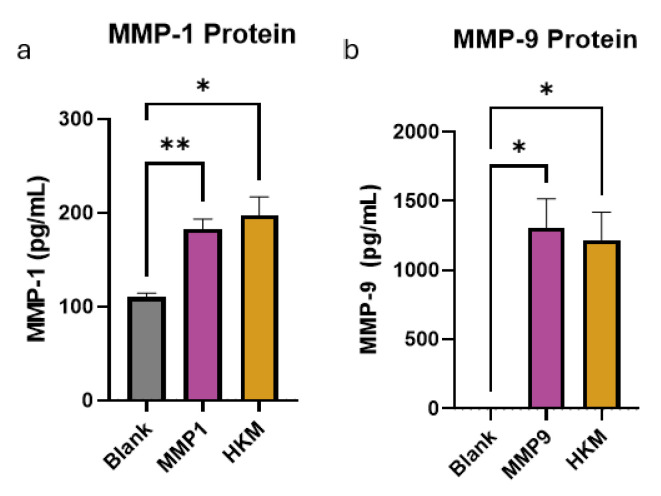
Human keratin matrix (HKM) does not reduce matrix metalloproteinase (MMP) activity by reduction in protein levels. (**a**) A bar graph showing MMP-1 protein levels after incubation with HKM as determined by ELISA. (**b**) A bar graph showing MMP-9 protein levels after incubation with HKM as determined by ELISA. Bars represent mean ± standard deviation. * *p* < 0.05, ** *p* < 0.01 by Brown–Forsythe and Welch analysis of variance (ANOVA) with Dunnet’s multiple comparisons for n = 3 independent replicates.

We next sought to identify the mechanism by which this reduction in MMP activity occurs. To determine if the enzyme was physically sequestered by the materials or deactivated in some other way, total protein levels in the solution were also measured by ELISA after incubation with HKM. In both MMP-1 and MMP-9, the total amount of protein was not different in solutions incubated with or without HKM (Figure 2). These data suggest that MMPs are not being sequestered from solution within the HKM, requiring further study of MMP activation and inhibition mechanisms.

### 2.3. HKM Reduces Zn^2+^ Ion Levels in Solution

**Figure 3:**
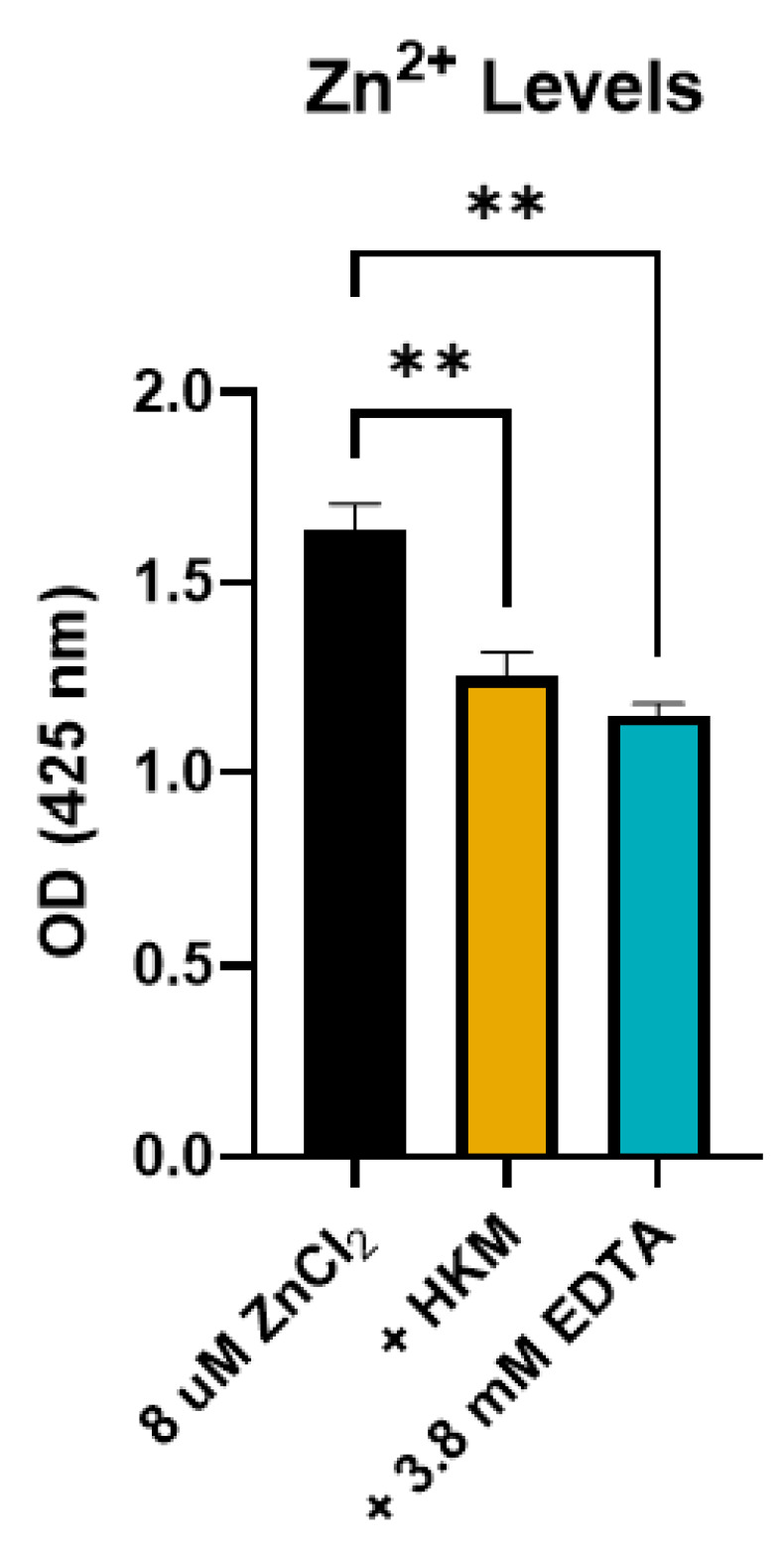
Human keratin matrix (HKM) reduces Zn^2+^ ion concentration in solution. Bar graph shows the concentration of zinc ions in solution after incubation with HKM compared with metal ion chelator ethylenediaminetetraacetic acid (EDTA). Bars represent mean ± standard deviation for n = 3 independent replicates. ** *p* < 0.01 by one-way analysis of variance (ANOVA) with Tukey’s multiple comparisons.

MMPs are dependent on coordination with a metal ion—namely zinc—for their proteolytic activity. Since HKM does not reduce the activity of MMPs by physically sequestering the enzymes, it was hypothesized that the keratin in HKM is preventing the activation of the enzymes. Because keratin is high in cysteine amino acids which are known to coordinate with zinc ions [19], we tested if HKM would reduce Zn^2+^ concentration in solution. An aqueous solution of zinc chloride was incubated with HKM or ethylenediaminetetraacetic acid (EDTA), a known chelator of metal ions. HKM significantly reduced the amount of zinc present in the solution, to a level statistically similar to EDTA, further suggesting keratin-mediated zinc chelation (Figure 3).

### 2.4. HKM Does Not Degrade nor Lose Efficacy in the Wound Environment

We next looked to a more clinically relevant application of these data: the longevity of HKM in the wound environment. Human keratin, like other advanced wound care products, is typically reapplied weekly in clinical settings and has been successful in closing wounds [20]. However, the high relative cost of these products has led to a recent interest in reducing the number of applications required to close a wound. Having shown HKM can reduce the activity of wound enzymes and is not degraded on short timescales by MMP-9, and given the known degradation resistance of other animal keratins [12], we tested if HKM would remain in place and maintain its healing efficacy with bi-weekly applications, compared to weekly applications in a diabetic mouse model of delayed wound healing.

**Figure 4:**
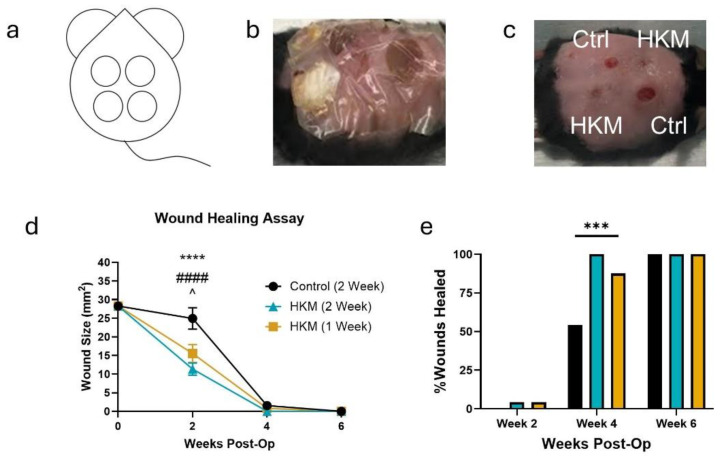
Human keratin matrix (HKM) maintains healing efficacy for at least two weeks in a diabetic mouse wound model. (**a**) Diagram of mouse with 4 dorsal wounds each treated with 2 control and 2 HKM in randomized locations. (**b**) Picture of a mouse with wounds dressed. (**c**) Representative photo of a mouse at week 4, showing HKM wound closed and control wound still open. (**d**) Graph of wound size over time for each treatment: control (black, circle), HKM changed bi-weekly (blue, triangle). HKM is changed weekly (gold, square) and is reprinted with permission from previous work for comparison under the Creative Commons Attribution license [19]. Points represent the mean; error bars represent the standard error of the mean for n = 12 mice. **** *p* < 0.0001 (control vs. HKM 2 Week), #### *p* < 0.0001 (Control vs. HKM 1 Week), ^ *p* < 0.05 (HKM 2 Week vs. HKM 1 Week) by two-way analysis of variance (ANOVA) with Tukey’s multiple comparisons. (**e**) Graph of the percentage of wounds healed for each treatment group at each timepoint (control—black, HKM changed weekly—gold [19], HKM changed bi-weekly—blue). *** *p* < 0.001 by Fisher’s exact test.

Four full-thickness wounds on the mouse dorsum were selected randomly for treatment with control dressings or HKM, and each mouse had dressings re-applied bi-weekly (Figure 4a,b). Wounds with weekly (previously published data included for comparison [15]) or bi-weekly reapplications of HKM showed faster wound closure (Figure 4c,d), and HKM-treated wounds were the first to close in this study (Figure 4e). A two-way ANOVA showed statistically significant differences by both treatment type (*p* = 0.0004) and time (*p* < 0.0001), and Sidak’s multiple comparisons between groups showed weekly and bi-weekly HKM treatments were statistically different (*p* = 0.0226 and *p* = 0.0003, respectively) from the control treatment but not from each other (*p* = 0.4420). Bi-weekly HKM wounds were all closed by week 4, and all wounds had closed by week 6 post-wounding (Figure 4e). These results show HKM is not degraded with two weeks of wound “wear time” and maintains its efficacy.

## 3. Discussion

In this work, we investigated two MMPs—a collagenase MMP-1 and a gelatinase MMP-9—and one serine protease (NE) to probe if HKM affects different wound enzymes. Interestingly, NE activity was not affected by HKM in vitro. Though elevated NE is predictive of wound nonhealing [8], increasing evidence demonstrates the importance of neutrophils and their neutrophil-derived proteases in wound healing [5]. Though this serine protease was not inhibited by the presence of keratin, other work has shown that keratin biomaterials may be effective delivery devices for specific elastase inhibitors [21]. Future work could investigate the use of HKM as a delivery device for other molecules to confer advanced functionality in the wound.

HKM did suppress the activity of MMPs, but this effect was not uniform between the MMPs studied despite the observed reduction in Zn^2+^ ions in the solution. Though the mechanism of the suppression of MMP-9 compared to MMP-1 requires additional study, there are subtle structural differences between these enzymes that may contribute to differences in inhibition. One such difference is the size of the peptide “pockets” that flank the metal ion in the catalytic domain of the enzyme [22]. MMP-1 has a smaller “pocket” domain than MMP-9, which has been leveraged in the development of selective MMP inhibitors [23]. Larger molecules can fit into the catalytic site pocket of MMP-9 but not MMP-1 [24], so the crosslinked keratin in HKM may have better access to inhibit MMP-9.

MMP-1 and MMP-9 are also distinct in their substrates as well as the cell types that produce them in the body, making their selective inhibition of interest in wound healing. MMP-1 is primarily produced by fibroblasts in the wound and specifically breaks down type I collagen [25]. While MMP-1 has long been known to be elevated in chronic wounds [10,26], it is also been shown more recently to be crucial in the wound-healing process [27]. In rats, the addition of MMP-1 during the healing process also reduced scarring [28], indicative of tissue regeneration rather than just wound closure. MMP-1 expression is also downregulated with increasing re-epithelialization [29] and wound closure. Because HKM accelerates wound closure, it may regulate MMP-1 activity and expression in other ways in vivo or clinically that were not reflected here in vitro.

MMP-9, however, can be produced by multiple cell types including immune cells or vascular endothelial cells [30], and has multiple substrates besides collagen that are important in mature extracellular matrix formation (e.g., fibronectin, laminin) [11]. This prevents new tissue from forming in the wound bed, and excessive MMP-9 in wound fluid has been shown to be a predictor of wound chronicity [8,9,31]. Here, we found that incubation with HKM in vitro reduces MMP-9 activity, suggesting that HKM may be a promising treatment for chronic wounds.

Interestingly, both MMPs and their inhibitors are demonstrated to be crucial to wound healing [32]. For example, MMP-9 knockouts significantly impair wound healing in mice and result in aberrant matrix production [33]. In the present work, MMP activity was assayed in an isolated system with just the enzyme, substrate, and HKM. However, in the wound, there are multiple cell types and biological factors, such as wound fluid that may be continually supplying the area with zinc ions or producing additional enzymes such that the MMP activity may be reduced but not to the levels shown here in vitro. The phase of wound healing and type of wound may also be important, as here we model delayed wound healing in vivo in a diabetic mouse. Elevated protease activity is associated with a prolonged inflammatory phase of wound healing, where healing may become stalled [34]. This highlights the complexity of the wound-healing process and further supports the increasing evidence that unbalanced protease activity is a major contributor to wound chronicity [35].

Similarly, zinc is also necessary for wound healing. Products containing zinc have long been used successfully in wound care, due in part to the support of normal metalloproteinase activity in the wound [36]. Even in chronic wounds, such as venous leg ulcers, zinc-containing compression wraps have shown benefit and are even considered standard of care. However, one recent case series of venous ulcer patients whose wounds did not respond to this standard treatment showed that the application of HKM significantly reduced wound size in these patients [18]. These results show the complexity and heterogeneity of wounds, and suggest that some wounds may benefit from different levels of zinc due to additional factors that require further study.

There are many other proteases present in the chronic wound environment, and additional studies could investigate if the present findings are broadly applicable to other MMPs and serine proteases. It is expected that the activity of other MMPs may be similarly suppressed through the metal ion chelation process described above. Other metalloenzymes could also be impacted by the presence of keratin, such as a disintegrin and metalloproteinase (ADAM) membrane-bound metalloproteinases. ADAMs are known to be upregulated in chronic wounds, and ADAM knockout mice demonstrate increased re-epithelialization [37,38]. As these enzymes are also zinc-binding, their interaction with HKM should be studied in subsequent work to continue to elucidate the role of HKM in wound healing. Additionally, one limitation of this work is that specific keratin subtypes were not evaluated for their role in the observed zinc level reduction. There are many keratin subtypes present in HKM [15]. Zinc binding may be due to high cysteine amino acid content [19], characteristic of the trichocytic or “hard” keratins found in hair [39], future work could further investigate if specific keratins are involved to develop more specific therapeutics for wounds.

In this work and elsewhere [15], we demonstrated treatment with HKM improves the delayed healing response of wounds in the db/db diabetic mouse model. Changes in protease activity were not directly assayed in vivo, another important limitation of this work, and only wound closure was quantified in the healing assay. Though the different phases of wound healing were not studied here, the importance of HKMs interaction with proteases throughout the healing process could be investigated further to better understand the role of human keratin in wound healing.

However, HKM remained as effective in closing wounds when left in place for two weeks compared to one week at a time, and both treatments showed significantly faster healing than control-treated wounds. This suggests HKM continues to be effective in the wound bed over these time periods, and keratin is not being removed from the wound area. Recent clinical work also supports these results. In the treatment of diabetic foot ulcers, a type of chronic wound characterized by elevated protease activity, a bi-weekly HKM replacement cycle was as effective as a weekly cycle in closing wounds [20]. Though additional work is needed to support HKM’s suppression of enzyme activity clinically, these results taken together show that HKM is a durable novel biomaterial for the treatment of chronic wounds.

In conclusion, this study demonstrated that HKM can play an important role in modulating the wound environment and supporting healing. HKM reduces the enzymatic activity of MMPs through the binding of Zn^2+^ ions in solution, which has clinical significance in the treatment of chronic wounds with elevated protease activity. This is the first published study demonstrating that HKM modulates the wound microenvironment in a way that may support wound healing, and opens the door for future study on the role of keratin in wound healing on a molecular level.

## 4. Materials and Methods

### 4.1. Preparation of HKM

HKM samples were produced as described previously [15]. Briefly, donated human hair was washed, delipidated, and air-dried prior to protein extraction in a custom Soxhlet (Chemglass Life Sciences, Vineland, NJ, USA). Dried hair was then extracted in a solution of urea, thiourea, and β-mercaptoethanol (Sigma-Aldrich, St. Louis, MO, USA) at 50 °C. The hair protein extract was then concentrated in stirred cells (Amicon, Millipore Sigma, Burlington, MA, USA) and cast into custom silicone molds that were air-dried in a fume hood until solid. Molds were then cured in 100% ethanol overnight at room temperature, partially rehydrated in 50% ethanol for 2 h, then washed extensively in type 3 water at 4 °C. Samples were then packaged in 50% propylene glycol (Sigma-Aldrich) in water to maintain moisture and sterilized by two doses of electron beam radiation (10.0 ± 1.0 kGy and 5.4 ± 0.5 kGy).

### 4.2. Enzymatic Activity Assays

The activity of MMP-1 and MMP-9 was measured using colorimetric enzyme inhibitor screening assay kits (Abcam, Waltham, MA, USA). NE activity was measured using a colorimetric drug discovery kit (Enzo Life Sciences, Farmingdale, NY, USA).

Samples of the enzyme were prepared at a concentration reflecting reported levels of test enzymes in chronic wound fluid: 600 ng/mL MMP-1 [40], 13.6 U/mL MMP-9 [8], or 23.64 mU/mL NE [8]. Samples of HKM were cut to 1 cm^2^ and incubated for 1 h in 1 mL of enzyme solution. As a control, enzyme inhibitors provided with kits were also incubated with the enzyme solutions for 1 h: 1.3 µM NNGH for MMPs and 100 µM elastatinal for NE. Subsequently, enzyme substrate was added to each sample and absorbance was read continuously at 1 min for 10 min in a colorimetric microplate reader (800TS, BioTek, Winooski, VT, USA). Reaction velocity was calculated for each enzyme by fitting a line to the linear region of absorbance over time data for each sample.

### 4.3. ELISA

Enzyme samples of MMP-1 and MMP-9 were prepared at clinically relevant concentrations and incubated with 1 cm^2^ HKM for 1 h, as described above. MMP levels were assayed using human MMP-9 and MMP-1 DuoSet ELISA kits (R&D Systems, Minneapolis, MN, USA), according to the manufacturer’s instructions. Absorbance was read with a colorimetric microplate reader (800TS, BioTek, Winooski, VT, USA).

### 4.4. Zinc Assay

Zinc levels in the solution were measured using a QuantiChrom^TM^ Zinc Assay Kit (BioAssay Systems, Hayward, CA, USA). A test solution of 8 µM ZnCl_2_ (Sigma-Aldrich) in water was prepared and then incubated with 1 cm^2^ samples of HKM or 3.8 mM EDTA for 1 h. Samples were then transferred to plastic cuvettes and absorbance at 425 nm was read with a UV-vis spectrophotometer (Vinmax 721 series, Amazon, Seattle, WA, USA).

### 4.5. In Vivo Wound Healing Model

The in vivo work in this study was approved by the Animal Care and Use Committee at Georgia State University in Atlanta, GA, USA. Animals were single-housed and given food and water ad libitum in sterilized cages in temperature-controlled rooms with a 12 h light–dark cycle. Twelve female db/db mice (BKS.Cg-Dock7^m^+/+Lepr^db^/J, 8 weeks old, Jackson Laboratory, Bar Harbour, ME, USA) had hair removed from the dorsum by shaving 3 days prior to surgery. Only female mice were used in order to facilitate group housing during the pre-experimental period, providing additional social benefits to the animals and reducing the risk of fighting and injuries that may confound the wound healing study. Mice were anesthetized with isoflurane using a precision vaporizer (World Precision Instruments, Sarasota, FL, USA), and four full-thickness circular wounds were created in the skin with a sterile 6 mm biopsy punch (Integra Life Sciences, Princeton, NJ, USA). Each wound was subsequently dressed with either HKM or no treatment (control) in randomized locations to account for potential variations in the healing rate of more cranial versus more caudal wounds for a total of 24 wounds of each treatment type. Each dressing was topped with cadexomer-iodine gel (Iodosorb, Smith and Nephew, London, England, UK), wound hydrogel (Skintegrity, Medline, Northfield, IL, USA), sterile gauze pads, and Tegaderm film (3M, St. Paul, MN, USA). Because mice tended to remove their dressing within the 2-week intervals, a staple was placed on the cranial and caudal end of the Tegaderm to secure the dressings in place.

At bi-weekly intervals (±1 day), wounds were debrided and measured, and fresh dressings were applied. This was chosen, as compared to previous studies with weekly wound measurements, to limit the risk of contamination from the removal when the same piece of HKM was reapplied for the second week. Wound area was calculated as the area of an ellipse using the longest and shortest measurements across the wound as the radii, and averaged by mouse for a total of n = 12 wound measurements for each group. Analgesia was provided by intraperitoneal injection of carprofen (5 mg/kg, Rimadyl, Zoetis, Parsippany-Troy Hills, NJ, USA) immediately following surgery, one day after surgery, and after each dressing change.

### 4.6. Statistical Analysis

Statistical analysis was performed using GraphPad Prism version 10.0.0 for Windows (Graph Pad Software, Boston, MA, USA). Enzyme activity and protein levels were analyzed with the Brown–Forsythe and Welch analysis of variance (ANOVA) with Dunnett’s multiple comparisons due to non-equivalent standard deviations. Zinc levels were compared with a one-way ANOVA with Tukey’s multiple comparisons. Wound size over time was analyzed with a two-way ANOVA with Tukey’s multiple comparisons. Percent healed was compared using Fisher’s exact test for proportions. Data in bar graphs are represented as mean ± standard deviation from triplicate samples unless otherwise noted. An alpha value of 0.05 was used as the threshold for significance in statistical testing.

## Data Availability

All non-proprietary data are contained within the article.
